# Salivary alpha amylase not chromogranin A reflects sympathetic activity: exercise responses in elite male wheelchair athletes with or without cervical spinal cord injury

**DOI:** 10.1186/s40798-016-0068-6

**Published:** 2017-01-04

**Authors:** Christof A. Leicht, Thomas A. W. Paulson, Victoria L. Goosey-Tolfrey, Nicolette C. Bishop

**Affiliations:** School of Sport, Exercise, and Health Sciences, The Peter Harrison Centre for Disability Sport, The National Centre for Sport and Exercise Medicine, Loughborough University, Loughborough, LE11 3TU UK

**Keywords:** Adrenaline, Catecholamines, Cortisol, Sympathetic dysfunction, Testosterone, Wheelchair athlete, Wheelchair propulsion

## Abstract

**Background:**

Salivary alpha amylase (sAA) and chromogranin A (sCgA) have both been suggested as non-invasive markers for sympathetic nervous system (SNS) activity. A complete cervical spinal cord injury leading to tetraplegia is accompanied with sympathetic dysfunction; the aim of this study was to establish the exercise response of these markers in this in vivo model.

**Methods:**

Twenty-six elite male wheelchair athletes (C6–C7 tetraplegia: *N* = 8, T6–L1 paraplegia: *N* = 10 and non-spinal cord injured controls: *N* = 8) performed treadmill exercise to exhaustion. Saliva and blood samples were taken pre, post and 30 min post exercise and analysed for sAA, sCgA and plasma adrenaline concentration, respectively.

**Results:**

In all three subgroups, sAA and sCgA were elevated post exercise (*P* < 0.05). Whilst sCgA was not different between subgroups, a group × time interaction for sAA explained the reduced post-exercise sAA activity in tetraplegia (162 ± 127 vs 313 ± 99 (paraplegia) and 328 ± 131 U mL^−1^ (controls), *P* = 0.005). The post-exercise increase in adrenaline was not apparent in tetraplegia (*P* = 0.74). A significant correlation was found between adrenaline and sAA (*r* = 0.60, *P* = 0.01), but not between adrenaline and sCgA (*r* = 0.06, *P* = 0.79).

**Conclusions:**

The blunted post-exercise rise in sAA and adrenaline in tetraplegia implies that both reflect SNS activity to some degree. It is questionable whether sCgA should be used as a marker for SNS activity, both due to the exercise response which is not different between the subgroups and its non-significant relationship with adrenaline.

## Key points

- This study shows a blunted alpha amylase but a normal chromogranin A response to maximal exercise in athletes with sympathetic dysfunction.
- This study would favour the use of alpha amylase over chromogranin A as a surrogate marker for sympathetic activity.
- However, in contrast to the adrenaline response, the alpha amylase response is not completely absent in cervical spinal cord injury, implying it is also regulated by mechanisms other than sympathetic activity.

## Background

Salivary secretions provide a non-invasive alternative to blood-derived markers to quantify exercise stress in both clinical and sports performance settings. Whilst salivary catecholamines have been suggested to be poor markers of acute sympathetic nervous system (SNS) activity [1], the salivary proteins α-amylase (sAA) and chromogranin A (sCgA) have been proposed to serve this purpose [2, 3]. Indeed, sAA activity is responsive to exercise, particularly that of a high-intensity nature, supporting the relationship between sAA and SNS activity [4–7]. Further, adrenergic receptor blockade can inhibit the stress-induced secretion of sAA [8]. However, the correlations between sAA and catecholamines in the circulation are relatively small [9, 10]. It has hence been questioned whether sAA truly reflects SNS activity as the influence of parasympathetic activity on sAA secretion may confound a stronger relationship [9, 11].

Chromogranin A is stored and co-released with adrenaline and noradrenaline from secretory vesicles within the adrenal medulla and post-ganglionic sympathetic axons [12]. It is also secreted from the submandibular gland following stimulation by noradrenaline and acetylcholine [13]. In response to acute exercise stress, sCgA shows a similar response to sAA [5, 14]. However, the correlation to physiological responses during acute exercise has been shown to be stronger for sCgA than for sAA [2]. This suggests a differential regulation of the two proteins and that sCgA may provide a more accurate representation of SNS activity. Conversely, non-exercise stress in the form of noise [15] or delivering a lecture [16] has been shown to increase sAA, whereas sCgA remained unaffected, questioning to what extent sCgA is regulated by the SNS.

Cortisol and testosterone represent two further markers of exercise stress and recovery that can be measured in plasma as well as in saliva. In response to acute exercise, both markers increase in a time and intensity-dependent manner [17, 18]. Cortisol and testosterone, expressed as a ratio, have also been put forward to describe the anabolic-catabolic balance. In this context, they may help in the diagnosis of the overtraining syndrome, even though it has been made clear that supporting markers are needed to define this condition [19].

The primary aim of this study was to investigate the impact of exercise to exhaustion on established plasma markers for SNS activity (adrenaline) and on proposed salivary markers for SNS activity (sAA and sCgA) in a human in vivo model of SNS dysfunction. The model of spinal cord injury (SCI) was employed: A complete injury to the cervical region of the spinal cord results in a tetraplegia (TETRA) and the dysfunction of the SNS, as evidenced by reduced cardiac acceleration [20] or a reduced catecholamine concentration at rest or following exercise [21]. However, increases in sAA activity have been found previously in both cervical- and thoracic-level athletes with SCI and non-spinal injured controls following strenuous exercise, which further questions sAA as a true indicator of central sympathetic drive [22]. We hence hypothesise the exercise-induced changes in adrenaline to be more closely related to sCgA than to sAA.

The secondary aim of this study was to investigate the impact of exercise on plasma and salivary steroid hormones in the SCI model. Whilst cortisol secretion is governed by humoral mechanisms (the hypothalamus-pituitary-adrenal or HPA axis), testosterone secretion is partly governed by neural pathways [23], explaining the high proportion of testosterone deficiency in the SCI population [24]. We hence hypothesise a normal plasma and salivary cortisol but a blunted testosterone response to exercise in TETRA.

## Methods

### Participants

The present data have not been previously published, but the samples were collected as part of a larger study [21]; as such, the participants studied and the exercise protocol employed are identical to this publication. Twenty-six international-level male wheelchair athletes volunteered to participate and were grouped according to their disability, TETRA, (*N* = 8), paraplegia (PARA, *N* = 10) and disabilities unrelated to SCI (non-spinal injured (NON-SCI), *N* = 8). All participants with SCI had a motor and sensory complete lesion in accordance with the American Spinal Injury Association (ASIA) impairment scale [25]. A summary of the participants’ characteristics and their main peak responses to exercise are presented in Table 1. All procedures were approved by the Loughborough University Ethical Advisory Committee and were in accordance with the Declaration of Helsinki. Participants provided written informed consent prior to the experiments, and they were free from infectious symptoms and pressure sores.

**Table 1:** Participants’ characteristics and peak exercise responses

	TETRA	PARA	NON-SCI
Age (years)	31 ± 6	30 ± 8	27 ± 8
Body mass (kg)	67.3 ± 5.9^a^	72.3 ± 13.5	84.8 ± 10.7
Disability	SCI C6–7	SCI T6–L1/spina bifida	Amputation/club foot
ASIA impairment scale	A	A	N/A
Wheelchair sport	Rugby	Basketball	Basketball
Training (h week^−1^)	13 ± 2	15 ± 3	16 ± 2
$\overset{.}{\mathrm{V}}{\mathrm{O}}_{2\mathrm{peak}}$ (L min^−1^)	1.44 ± 0.32^b^	2.85 ± 0.87^b^	3.75 ± 0.33^b^
HR_peak_ (b min^−1^)	127 ± 10^b^	181 ± 10	183 ± 8
BLa_peak_ (mmol L^−1^)	5.39 ± 0.96^b^	7.69 ± 1.87	8.29 ± 1.64
RPE_peak_	20 (19–20)	19 (19–20)	19 (19–20)

Data are mean ± SD or median (interquartile range); *P* < 0.05

*TETRA* tetraplegia, *PARA* paraplegia, *NON-SCI* non-spinal injured, *ASIA* American Spinal Injury Association, *HR* heart rate, *BLa* blood lactate concentration, *RPE* rating of perceived exertion, *SCI* spinal cord injury

^a^Significantly different from NON-SCI

^b^Significantly different from other subgroups

### Experimental protocol

Participants reported to the laboratory between 09:30 and 11:30 having been fasted for at least 2 h. They were asked to refrain from strenuous physical activity and caffeine intake 24 h prior to exercise. On arrival, their body mass was obtained to the nearest 0.1 kg using double-beam seated scales (Marsden MPWS-300, Rotherham, UK). All exercise tests were performed in the participants’ competition court sports wheelchair on a motorised treadmill (HP Cosmos, Traunstein, Germany) as described previously [21]. First, a ~30-min warm-up at intensities covering a range from 40 to 80% peak oxygen uptake $\left(\overset{.}{\mathrm{V}}{\mathrm{O}}_{2\mathrm{peak}}\right)$ was performed, followed by a 15-min passive recovery. A graded exercise test to exhaustion (GXT) was then completed at a constant speed. The gradient at the start of the GXT was 1.0% for all subgroups, with subsequent increases of 0.3% every minute for PARA and NON-SCI and 0.1% every 40 s for TETRA to account for the functional differences between subgroups and ensure a minimum GXT duration of ~8 min. After the GXT, participants recovered actively at a low intensity (1.2 m s^−1^ at a 1.0% gradient) for 5 min. Participants then performed a verification test, designed as a test to exhaustion at the same constant speed but 0.3 and 0.1% higher than the maximal gradient achieved during the GXT for NON-SCI/PARA and TETRA, respectively. The GXT and the verification test were terminated when participants were unable to maintain the speed of the treadmill. Verbal encouragement was given throughout the test, and participants were allowed to consume water ad libitum during the procedure.

### Data collection

Expired air was collected for at least the final three consecutive minutes of the GXT and for 2 min during the verification test and analysed using the Douglas bag technique, using a gas analyser (Series 1400; Servomex Ltd, Sussex, UK) and a dry gas meter (Harvard Apparatus, Kent, UK). Blood lactate concentration (BLa) was determined using a calibrated lactate analyser (YSI 1500 SPORT, YSI Incorporated, Yellow Springs, OH, USA) from a capillary blood sample obtained immediately after the GXT and immediately after the verification test. At the same time points, participants were asked to indicate an overall rating of perceived exertion (RPE) using the 15-point Borg scale according to previous instructions. Heart rate was continuously recorded at 5-s intervals (Polar PE 4000, Polar, Kempele, Finland). The higher of the two $\overset{.}{\mathrm{V}}{\mathrm{O}}_{2\mathrm{peak}},\ \mathrm{H}{\mathrm{R}}_{\mathrm{peak}}\kern0.5em \mathrm{and}\kern0.5em \mathrm{B}\mathrm{L}{\mathrm{a}}_{\mathrm{peak}}$ values obtained in the GXT and the verification test was taken as peak value. All participants had prior experience of the physiological testing procedure and were therefore familiar with the protocol.

Blood and saliva samples were collected before (pre), immediately after the verification test (post) and 30 min after exercise (post30). A 4.9-mL blood sample was drawn from an antecubital vein into a K_3_EDTA vacutainer. Timed, unstimulated saliva samples were collected as described previously [22]; the participants’ head slightly tilted forward with minimal orofacial movement during collection after rinsing their mouth out with water immediately before collection. Participants were allowed to consume water ad libitum apart from 6 min before each collection. Saliva flow rate was calculated by dividing the obtained saliva volume by the collection time.

### Plasma and saliva analysis

Blood samples were refrigerated until the final sample from each participant was collected and then centrifuged at 1500*g* for 10 min (at 4 °C). Whole saliva samples were immediately centrifuged at 13,000 rpm for 2 min following collection. The separated plasma was then immediately stored at −80 °C and saliva at −20 °C. Plasma concentrations of adrenaline, cortisol (pCort) and free testosterone (pTest) were determined using quantitative sandwich-type enzyme-linked immunosorbant assay (ELISA) kits (*adrenaline*: IBL international, Hamburg, Germany; *pCort and pTest*: DRG instruments, Marburg, Germany), according to the manufacturers’ instructions. All samples were analysed in duplicate. Saliva concentrations of cortisol (sCort), testosterone (sTest) and sCgA were also determined using ELISA (*sCort and sTest*: Salimetrics, Newmarket, UK; *sCgA*: Demeditic Diagnostics GmbH, Kiel, Germany) and sAA with an enzymatic activity assay as described previously [22]. All samples from one participant were analysed on the same ELISA plate; the within-assay coefficient of variation for the analyses performed was as follows: adrenaline 2.7%, pCort 1.3%, pTest 4.5%, sCort 2.8%, sTest 3.7%, sCgA 5.2% and sAA 4.9%.

### Statistical analysis

Data were analysed using IBM SPSS for Windows version 19 (SPSS inc, Chicago, IL). Exercise responses were analysed using a two-way mixed measures ANOVA with time as within- and group as between-measures variable for normally distributed variables, and significant main effects were assessed using Sidak post hoc tests. Adrenaline and sTest data were analysed using non-parametric Friedman tests for each group separately; adrenaline differences were assessed using Mann-Whitney *U* tests between TETRA and the other two subgroups (with intact sympathetic function) combined. Pearson-product moment correlations for normally distributed data and Spearman’s rho for non-normally distributed data were used to determine the relationship between plasma and salivary concentrations of cortisol and testosterone, as well as between adrenaline and the salivary proteins sAA and sCgA. Significance was set at *P* ≤ 0.05, and Bonferroni adjustments were performed when performing multiple comparisons. Data are presented as mean ± standard deviation.

## Results

**Fig. 1:**
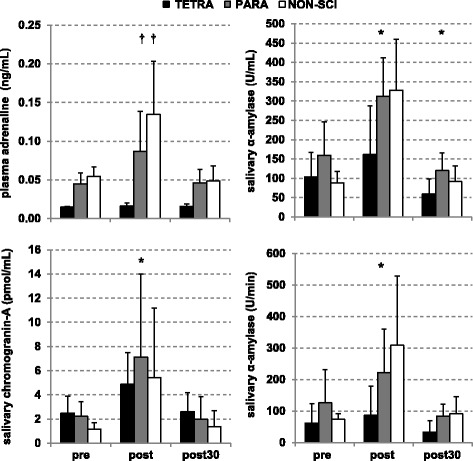
Plasma adrenaline, salivary α-amylase activity, chromogranin A and α-amylase secretion rate response to exhaustive exercise. *Daggers*: main effect of time for PARA and NON-SCI; *Asterisks*: significant difference from pre for all subgroups (*P* < 0.05)

A significant effect of time was found for plasma adrenaline concentration for both PARA and NON-SCI with a 2.2-fold increase from pre (*P* < 0.05) but not for TETRA (*P* = 0.74, Fig. 1, Table 2). Furthermore, the plasma adrenaline concentration was significantly lower in TETRA when compared with the other subgroups with intact sympathetic function (*P* < 0.001). The concentrations of sAA were highest at post exercise in all subgroups (2.3-fold increase from pre, *P* < 0.03), a main effect of group was found in sAA with lower values in TETRA than in PARA (*P* = 0.02) and a group × time interaction indicated a blunted response in TETRA (*P* = 0.005). The same pattern was found for the sAA secretion rate, with the exception that a main effect of group indicated lowest values for TETRA when compared with *both* other subgroups (*P* < 0.05). Post-exercise concentrations of sCgA were significantly elevated in all subgroups (threefold from pre, *P* < 0.001) with no difference between subgroups (*P* = 0.69). TETRA had a lower saliva flow rate than NON-SCI (*P* = 0.02), the saliva flow rate of PARA was not different from the other subgroups (*P* > 0.33). Saliva flow rate did not change over time (*P* = 0.77).

**Table 2:** Plasma and salivary responses to exhaustive exercise

Parameter (unit)	Time	TETRA	PARA	NON-SCI
Plasma adrenaline concentration (ng/mL)	Pre	0.02 ± 0.00	0.05 ± 0.01	0.05 ± 0.01
	Post	0.02 ± 0.00	0.09 ± 0.05^a^	0.14 ± 0.07^a^
	Post30	0.02 ± 0.00	0.05 ± 0.02	0.05 ± 0.02
Salivary chromogranin A concentration (pmol/mL)	Pre	2.47 ± 1.23	2.23 ± 1.22	1.18 ± 0.51
	Post	4.88 ± 2.63^a^	7.12 ± 6.88^a^	5.44 ± 5.75^a^
	Post30	2.61 ± 1.57	2.00 ± 1.87	1.37 ± 1.35
Salivary α-amylase activity (U/mL)	Pre	104 ± 63	160 ± 87	88 ± 30
	Post	162 ± 127^a^	313 ± 99^a^	328 ± 131^a^
	Post30	59 ± 39^a^	120 ± 46^a^	92 ± 40^a^
Salivary α-amylase secretion rate (U/min)	Pre	62 ± 62	126 ± 105	74 ± 18
	Post	86 ± 93^a^	222 ± 138^a^	309 ± 220^a^
	Post30	34 ± 36	84 ± 38	91 ± 54
Plasma cortisol concentration (ng/mL)	Pre	128 ± 13	113 ± 32	125 ± 33
	Post	178 ± 36^a^	184 ± 72^a^	169 ± 33^a^
	Post30	173 ± 64^a^	171 ± 58^a^	160 ± 47^a^
Salivary cortisol concentration (ng/mL)	Pre	5.48 ± 0.99	5.60 ± 1.75	5.46 ± 3.71
	Post	5.87 ± 1.66	6.05 ± 2.65	6.47 ± 3.71
	Post30	9.88 ± 6.24^ab^	11.69 ± 7.48^ab^	8.99 ± 4.23^ab^
Plasma testosterone concentration (ng/mL)	Pre	12.0 ± 4.6	10.9 ± 7.2	12.6 ± 5.4
	Post	15.8 ± 6.0	16.1 ± 17.4	15.5 ± 6.1
	Post30	15.4 ± 6.8	12.7 ± 12.7	11.6 ± 3.4
Salivary testosterone concentration (pg/mL)	Pre	128 ± 21	126 ± 32	107 ± 50
	Post	138 ± 15	131 ± 39	150 ± 36
	Post30	141 ± 23	122 ± 24	131 ± 33
Saliva flow rate (mL/min)	Pre	0.52 ± 0.22	0.78 ± 0.54	0.95 ± 0.43
	Post	0.45 ± 0.18	0.72 ± 0.40	0.95 ± 0.45
	Post30	0.51 ± 0.22	0.72 ± 0.27	0.99 ± 0.33

Data are mean ± SD; *P* < 0.05

^a^Significantly different from pre

^b^Significantly different from post

**Fig. 2:**
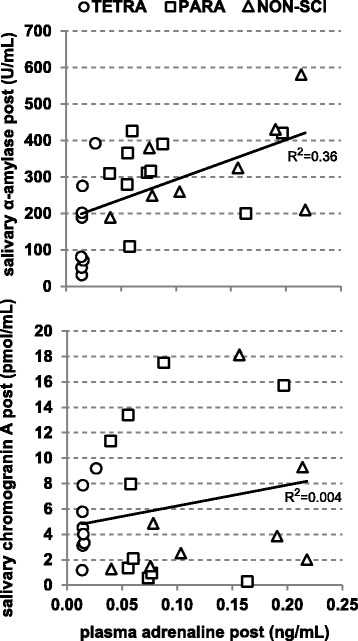
Suggested salivary markers for SNS activity and their relationship with plasma adrenaline after exhaustive exercise. *R* ^*2*^ coefficient of determination

Correlations between plasma adrenaline concentration and salivary parameter post exercise were significant for sAA (*r* = 0.60, *P* = 0.01), but not for sCgA (*r* = 0.06, *P* = 0.79, Fig. 2). The correlation between sAA and sCgA was also not significant (*r* = 0.25, *P* = 0.23).

**Fig. 3:**
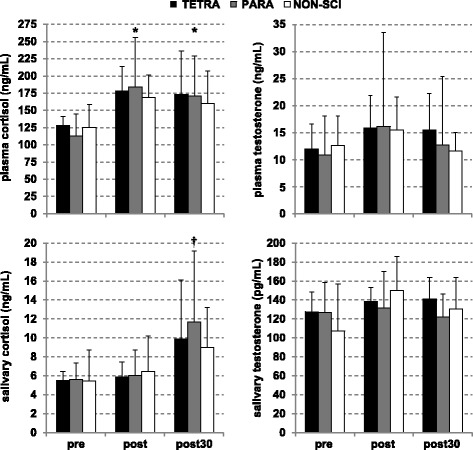
Cortisol and testosterone response to exhaustive exercise. *Asterisks*: significant difference from pre for all subgroups; *Dagger*: significant difference from pre and post for all subgroups (*P* < 0.05)

Plasma and salivary steroid concentrations are shown in Fig. 3 and in Table 2. Significant effects of time (*P* < 0.001) demonstrate an increase in both plasma and salivary cortisol concentrations following exercise, with the highest concentrations found at post and post30 for plasma (both 1.4-fold elevated from pre), whilst salivary concentrations were only elevated at post30 (1.9-fold from pre, *P* < 0.01). No significant group (*P* > 0.89) or interaction (*P* > 0.68) effects were found for plasma and salivary cortisol concentrations. A main effect of time was found for pTest (*P* = 0.02) which only showed a trend for post concentrations to be elevated from the other time points (1.3-fold from pre, *P* = 0.06), whereas no group or time effects were found for sTest (*P* > 0.05).

Significant correlations were found between plasma and salivary concentrations of cortisol both at pre (*r* = 0.60, *P* = 0.003) and post30 (*r* = 0.68, *P* < 0.001), whereas a trend was evident at post (*r* = 0.43, *P* = 0.09). In contrast, no significant correlation was found between corresponding plasma and salivary testosterone concentrations (pre: *r* = 0.08; post: *r* = 0.34; post30: *r* = 0.21; *P* > 0.05).

## Discussion

The main findings of the present study were that (1) both sAA and sCgA concentrations increased following exhaustive exercise in all spinal injury level subgroups; (2) the post-exercise sAA response was blunted in TETRA; (3) post-exercise plasma adrenaline concentrations were significantly correlated with post-exercise sAA concentrations, but not with sCgA concentrations; (4) in contrast to the other subgroups, the plasma adrenaline concentrations in TETRA did not alter in response to exercise; and (5) salivary cortisol but not testosterone reflected plasma activity, the responses not being different between subgroups.

### Are sAA and sCgA appropriate salivary markers for sympathetic activity?

The primary aim of this study was to investigate the impact of strenuous exercise on established plasma (adrenaline) and salivary (sAA and sCgA) markers for SNS activity in a human in vivo model of SNS dysfunction. The present results support the finding that sAA is partly governed by SNS activity [11], as a blunted response was observed in TETRA when compared with the other subgroups. The correlation between adrenaline and sAA further imply that sAA could be used as a surrogate marker for adrenaline. However, the variance explained was only 36% (*R* ^2^), leaving a considerable amount of unexplained random variation, which is of a similar magnitude as found previously [9]. It is likely that some of this random variation is explained by the contribution of the parasympathetic nervous system [11], which contributes to sAA, but not to adrenaline release. Therefore, whilst plasma catecholamine concentrations represent a gold standard with respect to SNS activity, sAA activity may be used to help indicating SNS dysfunction in an exercise context. These results are in contrast to earlier findings, where no significant differences between the same subgroups as investigated in the current study have been found with respect to sAA activity following strenuous interval exercise [22]. However, closer inspection of these previous data reveals a 22% lower sAA activity in TETRA post exercise, even though the difference to the subgroups with intact SNS was insignificant. It should further be noted that the exercise performed during this previous study was not performed to exhaustion as in the current study. This could further explain the previous non-significant difference, as the subgroups with intact SNS activity were likely not to initiate the full potential of their sAA response. Indeed, exercise of an incremental nature with continuous data sampling shows a continuous increase in sAA and sCgA concentration with increasing exercise intensity which led to the suggestion for their use as markers for exercise intensity [14].

Whilst sAA responds to stressors other than exercise, such as emotional stressors [9], it is worth noting that Bocanegra et al. [14] suggest sAA and sCgA as markers for *exercise intensity*, not *sympathetic activity* in exercising contexts. This is an aspect worth developing: correlation (between salivary markers and exercise intensity) does not necessarily imply causation (of their regulation by the SNS), as demonstrated by comparing the present findings with the data presented by Bocanegra et al. [14]. Despite the strong relationships between sAA, sCgA and exercise intensity [14], factors other than SNS activity, such as parasympathetic activity, reflex activity or, in the case of SCI, receptor hypersensitivity [26], may also contribute to the changes observed in these salivary markers in response to exercise. Therefore, SNS activity cannot be suggested as their main modulating component. This thought may be further developed to question the use of sCgA as a marker of SNS activity as previously suggested [2], as the present data do not support sCgA as a marker for SNS activity for two reasons: first, the exercise response is not different between the wheelchair athlete subgroups, and second, the relationship with adrenaline is insignificant.

### The steroid hormone and saliva flow rate response in SCI

The cortisol exercise response was not different between subgroups. This is consistent with the humoral regulation of cortisol release, which is independent of SNS function. In contrast, testosterone is secreted partly through the action of neuronal mechanisms [23], which again lends itself to be studied with the SCI model. However, no group or interaction effects were found for the testosterone responses, implying that testosterone regulation is only minimally, if at all, dependent on neural activity in the context of an exercise intervention.

The present cortisol data corroborate earlier results: A positive association between salivary and circulating plasma/serum cortisol concentrations has been confirmed both at rest [27–29] and following exercise [30–33]. Further, we have shown that exercise induces a rise in plasma cortisol concentration immediately following exercise, a response which can be observed with a time lag in the salivary concentrations. Similar effects have been found previously in direct comparisons of plasma and salivary cortisol concentrations [34], and exercise studies report that the sCort peak trails the pCort peak, which is usually found soon after cessation of exercise [33]. As sCort originates from the circulation, this lag is likely due to the process of cortisol diffusion and ultrafiltration through acinar cells [35], and the lag between pCort and sCort peak values has been reported to be in the area of ~10–20 min [33]. This is consistent with our findings—the weakest correlation between pCort and sCort was found post exercise, where the sCort concentration had not reached its maximum yet. It is therefore likely that the correlation between these two markers could be improved when comparing post-exercise pCort values with sCort sampled with a delay of 10–20 min. Including a sampling lag for sCort may also be relevant whenever the focus lies on determining the true maximum cortisol value, for example in the context of quantifying acute exercise stress [33] or in the context of over-reaching [36].

A modest but significant increase was observed in pTest, but not in sTest for all subgroups. The increases in pTest are consistent with previous reports [33], therefore suggesting that pTest may be used in SCI subgroups for the same diagnostic purposes as in the able-bodied population. The poor relationships between salivary and plasma testosterone also support previous research that questions the diagnostic value of salivary testosterone [35]; even though significant relationships have been shown between salivary and plasma/serum concentrations [27, 32], this was not replicated by others [33]. From a clinical perspective, the present study does not provide any evidence for testosterone deficiency in TETRA as previously reported [24]. However, previous research shows that exercise training increases resting testosterone levels in chronic SCI [37]; the highly trained nature of the investigated participant group is hence a likely explanation of this finding.

Finally, the reductions in saliva flow rate in TETRA underline the physiological difference of this subgroup—saliva flow rate can be increased by adrenoreceptor agonists but decreased by adrenoreceptor blocking drugs; furthermore, it is affected by neural stimulation [38]. The chronically lower adrenaline concentrations and interruption of neural pathways to the salivary glands in TETRA are a likely reason for these observed reductions in saliva flow rate.

### Future directions

In addition to adrenaline, a number of studies report relationships between noradrenaline and suggested markers for SNS activity [3, 13]. Whilst noradrenaline was not measured in the present study, it is highly likely that the noradrenaline response was similarly blunted as the adrenaline response, as shown earlier for cervical SCI [39, 40]. Despite this, we suggest assessing the noradrenaline response in follow-up studies, which would allow a distinction between secretion mechanisms governed mainly by the adrenal glands (adrenaline) or secretion by sympathetic nerve endings (noradrenaline).

## Conclusions

The blunted post-exercise rise in sAA and adrenaline in TETRA implies that both reflect SNS activity to some degree. Even though the sAA response to exercise is not absent, as is the case for adrenaline, sAA appears to reflect some of the SNS dysfunction found in TETRA. Therefore, rather than describing sAA as a marker of SNS activity in its own right, it would be more accurate to refer to it as a marker which *partly* reflects SNS activity but is also regulated by other mechanisms. Despite this limitation, we suggest that to date, sAA is the best surrogate salivary marker for SNS activity, which is relevant in the absence of available blood tests and if restricted to salivary analyses. This is in contrast to earlier findings which have proposed that sCgA may provide a more accurate representation of SNS activity than sAA [2] but supports the findings from non-exercise stress interventions that failed to observe increases in sCgA in the presence of sAA elevations [15, 16].

On a final note, plasma and salivary cortisol and testosterone responses to exercise did not differ between subgroups, implying a minimal involvement of sympathetic innervation in the acute exercise response.

## Abbreviations

BLa: Blood lactate concentration
GXT: Graded exercise test to exhaustion
HPA: Hypothalamus-pituitary-adrenal
HR: Heart rate
NON-SCI: Non-spinal injured
PARA: Paraplegia
pCort: Plasma cortisol
pTest: Plasma testosterone
RPE: Rating of perceived exertion
sAA: Salivary alpha amylase
sCgA: Salivary chromogranin A
SCI: Spinal cord injury
sCort: Salivary cortisol
SNS: Sympathetic nervous system
sTest: Salivary testosterone
TETRA: Tetraplegia
